# Lung injury-dependent oxidative status and chymotrypsin-like activity of skeletal muscles in hamsters with experimental emphysema

**DOI:** 10.1186/1471-2474-14-39

**Published:** 2013-01-23

**Authors:** Jair Tonon, Alessandra Lourenço Cecchini, Cláudia Roberta Brunnquell, Sara Santos Bernardes, Rubens Cecchini, Flávia Alessandra Guarnier

**Affiliations:** 1 Laboratory of Free Radicals and Pathophysiology, Department of General Pathology, Rodovia Celso Garcia Cid, PR445, km 380 Campus Universitário, Londrina 86051-990, Brazil; 2 Laboratory of Molecular Pathology, Department of General Pathology, Rodovia Celso Garcia Cid, PR445, km 380 Campus Universitário, Londrina, 86051-990, Brazil; 3 Laboratory of Free Radicals and Pathophysiology, Department of General Pathology, Rodovia Celso Garcia Cid, PR445, km 380 Campus Universitário, Londrina, 86051-990, Brazil; 4 Laboratory of Molecular Pathology, Department of General Pathology, Rodovia Celso Garcia Cid, PR445, km 380 Campus Universitário, Londrina, 86051-990, Brazil; 5 Laboratory of Free Radicals and Pathophysiology, Department of General Pathology, Rodovia Celso Garcia Cid, PR445, km 380 Campus Universitário, Londrina, 86051-990, Brazil; 6 Laboratory of Free Radicals and Pathophysiology, Department of General Pathology, Rodovia Celso Garcia Cid, PR445, km 380 Campus Universitário, Londrina 86051-990, Brazil

**Keywords:** Emphysema, Cachexia, Skeletal muscle loss, Reactive oxygen species, Chymotrypsin-like activity

## Abstract

**Background:**

Peripheral skeletal muscle is altered in patients suffering from emphysema and chronic obstructive pulmonary disease (COPD). Oxidative stress have been demonstrated to participate on skeletal muscle loss of several states, including disuse atrophy, mechanical ventilation, and chronic diseases. No evidences have demonstrated the occurance in a severity manner.

**Methods:**

We evaluated body weight, muscle loss, oxidative stress, and chymotrypsin-like proteolytic activity in the gastrocnemius muscle of emphysemic hamsters. The experimental animals had 2 different severities of lung damage from experimental emphysema induced by 20 mg/mL (E20) and 40 mg/mL (E40) papain.

**Results:**

The severity of emphysema increased significantly in E20 (60.52 ± 2.8, p < 0.05) and E40 (52.27 ± 4.7; crossed the alveolar intercepts) groups. As compared to the control group, there was a reduction on body (171.6 ± 15.9 g) and muscle weight (251.87 ± 24.87 mg) in the E20 group (157.5 ± 10.3 mg and 230.12 ± 23.52 mg, for body and muscle weight, respectively), which was accentuated in the E40 group (137.4 ± 7.2 g and 197.87 ± 10.49 mg, for body and muscle weight, respectively). Additionally, the thiobarbituric acid reactive substances (TBARS), tert-butyl hydroperoxide-initiated chemiluminescence (CL), carbonylated proteins, and chymotrypsin-like proteolytic activity were elevated in the E40 group as compared to the E20 group (p < 0.05 for all comparisons). The severity of emphysema significantly correlated with the progressive increase in CL (r = −0.95), TBARS (r = −0.98), carbonyl proteins (r = −0.99), and chymotrypsin-like proteolytic activity (r = −0.90). Furthermore, augmentation of proteolytic activity correlated significantly with CL (r = 0.97), TBARS (r = 0.96), and carbonyl proteins (r = 0.91).

**Conclusions:**

Taken together, the results of the present study suggest that muscle atrophy observed in this model of emphysema is mediated by increased muscle chymotrypsin-like activity, with possible involvement of oxidative stress in a severity-dependent manner.

## Background

Emphysema is a form of chronic obstructive pulmonary disease (COPD), which is associated with high morbidity and mortality worldwide. Emphysema is primarily caused by smoking, although environmental pollution and α1-antitrypsin deficiency may also lead to its development [1]. Patients typically present altered muscle mass and exercise intolerance [2], and this loss of skeletal muscle mass is a common observation in patients with COPD [1]. Skeletal muscle atrophy is an important systemic consequence, and reduced mid thigh muscle cross sectional area has a strong impact on mortality [3]. Although the biochemical pathways engaged in the development of muscle atrophy are poorly understood, an imbalance between protein breakdown and synthesis, in favor of the former, is believed to play a role in this process [4]. In addition to muscle weakness [5], human studies demonstrated that emphysema is also associated with reduced oxidative enzyme activities [6], and elevated exercise-induced muscle phosphocreatine activity [7]. Unfortunately, whether these alterations result from reduced physical activity or from COPD itself is yet to be determined in humans. In addition, data about extension of lung damage and extension of systemic manifestations are scarce. Experimental models of pulmonary emphysema using proteolytic enzymes such as papain or elastase, either instilled or nebulized into the airways of animals, are based on imbalanced protective and aggressive substances in pulmonary tissue. These models result in morphological and histological alterations equivalent to those in humans [8]. The inexpensive, papain-induced emphysema model results in pulmonary and systemic alterations characteristic of emphysema patients. Ventilatory mechanical alterations, increased residual functional capacity, total lung volume, pulmonary complacence [9], cardiac overload [10], and skeletal muscle mass loss [11] are previously reported emphysema-associated alterations.

Mattson and Poole [12] demonstrated that decreased citrate synthase activity in peripheral skeletal muscle of emphysemic hamsters was not associated with the level of animal activity. In addition, the same group of authors [2] reported increased lipid peroxidation, as assessed by malondyaldehyde (MDA) and glutathione levels in the skeletal muscle of hamsters with emphysema. Since the 1990s, investigators have demonstrated that patients suffering from emphysema, chronic bronchitis, and asthma have increased lipid peroxidation, a marker of oxidative stress [13]. Furthermore, previous studies have indicated that elevated levels of reactive oxygen species (ROS) may predispose muscle tissue to fatigue [14] and that ROS are signaling molecules involved in muscle adaptation; furthermore, redox-sensitive kinases, phosphatases, and nuclear factor-κB have been implicated in muscle loss [15]. Previous studies have described the connection between redox signaling and skeletal muscle adaptation in response to increased muscular activity (i.e., exercise training) [16,17] and prolonged periods of muscular inactivity (i.e., immobilization) [18]. Several lines of evidence indicate that ROS signaling is involved in the regulation of the ubiquitin–proteasome system. For example, oxidative stress has been shown to stimulate ubiquitin conjugation to muscle proteins through the transcriptional regulation of the enzymes (i.e., E2 and E3 proteins) that conjugate ubiquitin to muscle proteins to promote proteolysis [19]. In theory, increased expression of the E2 and E3 proteins in skeletal muscles would lead to accelerated proteolysis via the 26S proteasome. Furthermore, evidence indicates that the 20S (core) proteasome can degrade oxidized proteins without ubiquitination [20]. Therefore, it is likely that oxidative stress can accelerate muscle protein breakdown via both the 20S proteasome (alone) and the 26S proteasome complex [15]. The 26S proteasome is a 2.5-MDa multi-protein complex found in both the nucleus and cytosol of eukaryotic cells; it is comprised of a single 20S core particle and 19S regulatory particles at one or both ends. Three major proteolytic activities (described as chymotrypsin-like, trypsin-like, and post-glutamyl peptide hydrolytic or caspase-like activity) occur in the 20S core. Together, these 3 activities are responsible for most of the protein degradation required for maintaining cellular homeostasis, including degradation of damaged cellular proteins. This system is also involved in essential cellular processes such as the response to hypoxemia and muscle tissue regeneration. Existing evidence links the activity of the ubiquitin-proteasome system and the cellular events that occur in the respiratory and peripheral muscles of COPD patients [21]. Thus far, no studies have indicated whether proteolytic activity and oxidative stress are correlated with COPD severity.

To determine whether oxidative stress plays an important role in the regulation of skeletal muscle mass in a COPD model and if it is dependent of the severity, we evaluated body weight, muscle loss, oxidative stress, and chymotrypsin-like proteolytic activity in gastrocnemius muscle tissue in hamsters with 2 different severities of experimental emphysema. This paper reports that emphysema induces increased muscle loss in a way dependent on lung injury extension. In addition, oxidative stress, protein degradation, and chymotrypsin-like proteolytic activity seem to contribute to this mechanism.

## Methods

### Animals

Adult male Syrian Golden hamsters, weighing 130–150 g, were used (n = 8/group). The animals were given water and commercial food (Nuvilab CR1; Nuvital Nutrients Ltd., Curitiba, Brazil) *ad libitum*, and the environment was controlled on a 12-h light/dark cycle. The protocols conformed to the Guide for the Care and Use of Laboratory Animals [DHEW Publication No. (NIH) 86–23, Revised 1985, Office of Science and Health Reports, DRR/NIH, Bethesda, MD 20892], and the study was approved by Ethics Committee on Animal Experimentation from the Universidade Estadual de Londrina, Brazil (ref. 5500).

The animals were randomly divided into 3 groups, according to the instillation procedure and dose of papain used to induce emphysema. Under deep ketamine/xylazine anesthesia (150/30 mg/kg im), either saline (0.3 mL/100 g body weight) or papain (20 or 40 mg/100 g body weight [Viafarma, São Paulo, Brazil] in 0.3 mL of normal saline) was instilled intratracheally using a 27-gauge hypodermic needle, according to the procedure described by Mattson et al. [2] with modifications. The procedure consists on making a short cut on the skin of the tracheal region, seclusion of muscle layer and trachea, insertion of the needle between tracheal rings, instillation and final suture of the skin. To ensure a uniform papain distribution throughout the lungs, each hamster was submitted to a gentle manual negative pressure maneuver. Briefly, just after papain instillation, in the moment of final expiration, thorax was momently restrained in order to avoid lung expansion. After active inspiration, a negative pressure is generated and thorax released. This maneuver, through acute differences of pressure, allows complete spread of papain until distal airways. The 3 groups were labeled as follows: control + saline (CS), animals instilled with only approximately 0.3 mL of saline; emphysema 20 mg/mL (E20), animals instilled with approximately 0.3 mL of 20 mg/mL papain in saline; and emphysema 40 mg/mL (E40), animals instilled with approximately 0.3 mL of 40 mg/mL papain in saline. After surgery, the animals were returned to their cages; their appearance and body weights were monitored daily for the first 2 weeks and once a week thereafter, for 60 days.

### Tissue collection and preparation

Sixty days after papain injection, the hamsters were weighed and euthanized. The gastrocnemius muscle was excised, weighed, frozen in liquid nitrogen, and stored at −86°C until use. The gastrocnemius muscle has previously been demonstrated as a good indicator of alterations in the skeletal muscle of emphysemic hamsters [22], including those in lipid peroxidation [2]. In hamsters, there is no obvious gross division of fibers in this muscle [23], and therefore, the whole muscle is typically analyzed.

The middle lobe of the left lung was fixed in 10% formalin for morphometric evaluations. For the oxidative stress analysis, muscles from CS, E20, and E40 mice were prepared as described above. Tissues were placed on ice and homogenized for 60-s periods at 60-s intervals in an Ultraturrax homogenizer containing 10 mg/mL or 50 mg/mL of tissue in 10 mM KH_2_PO_4_/K_2_HPO_4_ buffer and 120 mM KCl at pH 7.4. The total homogenate (10 mg/mL) was used for the tert-butyl hydroperoxide-stimulated chemiluminescence (CL) and thiobarbituric acid reactive substances (TBARS) assays. For total protein carbonylation determination, tissues were treated according to the method of Reznick and Packer [24], with adaptations as described below. For chymotrypsin-like activity analysis, during muscle excision, a segment of the gastrocnemius muscle of each animal was frozen in liquid nitrogen, pulverized, and frozen at −86°C. Additionally, the cachexia index was determined (considering initial and final body weight of the emphysemic animals and body weight gain in the CS group) in order to identify a pattern of general wasting [25].

### Morphometric analysis

After the middle lobe of the right lung was removed, samples were fixed in paraformaldehyde for 48 h and embedded in paraffin for histological studies. Paraffin-embedded tissues were sectioned into ~5 μm per lung. Sections were stained with hematoxylin and eosin.

To verify emphysema establishment and severity, alveolar destruction was determined by the number of times that a predetermined group of coherent lines (1.25 mm^2^ of area and 1.50 mm of total length) crossed the parenchymal structures. The group identifications were covered, and lung images were captured (5 fields/section, 3 semi-serial sections/animal) using an optical microscope (50× magnification). Images were obtained using a high-resolution camera coupled to the microscope. The base lines were then superposed onto the images; the lesser the structures were crossed, the more extensive was the lesion [8]. An image analysis system (Image-Pro-Plus 4.0; Media Cybernetics, Silver Spring, MD, USA) was used to determine and count the number of intersections.

### Determination of TBARS

The extent of lipid peroxidation of the muscle homogenates from each group was determined by the TBARS reaction. MDA formed during peroxidation reacts in the TBA test to generate a colored product, a (TBA)_2_–MDA adduct. In an acid solution, (TBA)_2_–MDA absorbs light at 532 nm and is readily extractable by an organic solvent such as butanol. MDA levels were measured, and the results were expressed in nmol/g tissue, as described by Oliveira and Cecchini [26].

### Carbonyl protein content

The carbonyl protein content was measured as described by Reznick and Parker [24], with modifications. Approximately 200 mg of gastrocnemius muscle were placed in glass homogenization tubes containing 4 mL of homogenizing buffer (50 mM phosphate buffer, 1 mM Ethylenediamine tetraacetic acid, pH 7.4). Tissue samples were homogenized and incubated for 15 min in an ice bath. The samples were centrifuged at 3,000 × *g* for 10 min at room temperature (RT), and 1 mL of each protein extract was placed in glass tubes. A volume of 4 mL of 2,4-dinitrophenylhydrazine (DNPH) solution prepared in 2.5 N HCl was added to each tube, and the reaction mixtures were incubated for 1 h at RT, with vortexing every 15 min. Next, the samples were washed with 5 mL of 20% trichloroacetic acid (TCA) (w/v) and centrifuged for 10 min to collect the protein precipitates. Another wash was performed using 10% TCA, and protein pellets were dispersed mechanically. Finally, the pellets were washed 3 times with 4 mL of ethanol-ethyl acetate (1:1, v/v) to remove free DNPH and lipid contaminants. The final precipitates were dissolved in 2 mL of 6 M guanidine hydrochloride, and any insoluble materials were removed by additional centrifugation. The carbonyl content was calculated by reading the peak absorbance at 355–390 nm of the DNPH-treated samples, versus samples treated with only 2.5 M HCl. The following formula was used to calculate the concentration of carbonyls: C = Abs (355–390nm) × 45.45 nmol/mL, where C is the concentration of DNPH/mL, and 45.45 is its absorption coefficient. The procedures were performed in an ice bath until the TCA wash step. The carbonyl content was expressed as nmol/mg total protein.

### Measurement of tert-butyl hydroperoxide-initiated CL

Reaction mixtures were placed in 2-mL luminescence tubes containing the following: total muscle homogenate (8.75 mg/mL), 10 mM KH_2_PO_4_/K_2_HPO_4_ buffer (with 120 mM KCl, pH 7.4), and 6 mM tert-butyl hydroperoxide, in a final volume of 1 mL. The tert-butyl hydroperoxide-initiated CL reaction was assessed using a TD 20/20 luminometer (Turner Designs, Sunnyvale, CA, USA), with a response range of 300–650 nm. The tubes were kept in the dark until the assay was carried out in a room at 33°C [26,27]. For each animal, a 40-min curve, in which each point represented the differential smoothing of 600 readings, was obtained by interpolation. The results were expressed in relative light units/g tissue (RLU/g tissue); after the final calculation, the area, extracted by integral calculus of each animal curve, were used to determine the amount of lipid hydroperoxides present in the sample.

### Measurement of the total antioxidant capacity of muscle (TRAP)

Total antioxidant capacity of homogenates prepared as described before was measured by CL, in a reaction medium containing 20 μM 2-azo-bis-(2-amidinopropane) and 200 μM luminol. After maximal emission was attained, 70 μL of tissue supernatant or trolox were added to the reaction medium. The time of total quenching was compared with trolox quenching, and the results were expressed in μM trolox [28].

### Chymotrypsin-like activity

Chymotrypsin-like proteolytic activity was measured using a Proteasome Glo™ Chymiotrypsin-like Cell Based Assay kit (Promega, Madison, WI, USA). This kit estimates the activity of the 20S proteasome; the assay involves the use of a specific luminogenic substrate (succinyl-leucine-valine-tyrosine-aminoluciferin) to determine chymotrypsin-like activity. The proteasome cleavage of the substrate produces a luminescent signal by the luciferase contained in the reaction medium. The 3 major proteolytic activities (chymotrypsin-like, trypsin-like, and post-glutamyl peptide hydrolytic or caspase-like activity), occurring within the 20S core of the 26S proteasome complex are responsible for most of the protein degradation, which includes degradation of damaged cellular proteins. Therefore, this coupled-enzyme system, with simultaneous proteasome cleavage of substrate and luciferase consumption of the released aminoluciferin, results in a luminescent signal that can be considered proportional to the amount of proteasome activity in the muscle tissue.

During muscle excision, a segment of the gastrocnemius muscle of each animal was frozen in liquid nitrogen, pulverized, and frozen at −86°C. For the assay, 25 mg of the muscle powder was added to 1 mL of 10mM KH_2_PO_4_, pH 7.4, in 0.9% NaCl and gently homogenized. Fifty microliter of the resulting muscle homogenate was pipetted in duplicate onto 96-well microplates, and the final reagent mixture was added to the medium. After 5 minutes, under light protection, the plate was read. The luminescent signal was detected with a Glo-Runner microplate reader luminometer (Turner Designs), and the results were expressed as RLU/mg tissue.

### Protein concentration

The protein concentration was determined by the method of Lowry et al. [29], with modifications as described by Miller [30]. This method involved the use of bovine serum albumin (BSA) as a standard.

### Statistical analysis

The results are presented as mean ± standard error of the mean (SEM) for 8 animals. All values were compared using one-way analysis of variance followed by Bonferroni’s multiple comparison test, with p < 0.05 considered significant. To evaluate the correlation between oxidative variables and emphysema severity and between oxidative variables and chymotrypsin-like proteolytic activity, Mean, SE and N from each group were combined and submitted to the Pearson’s correlation test in order to establish the correlations between the variables. Values of p < 0.05 were considered significant for all analyses.

## Results

### Emphysema condition

**Figure 1:**
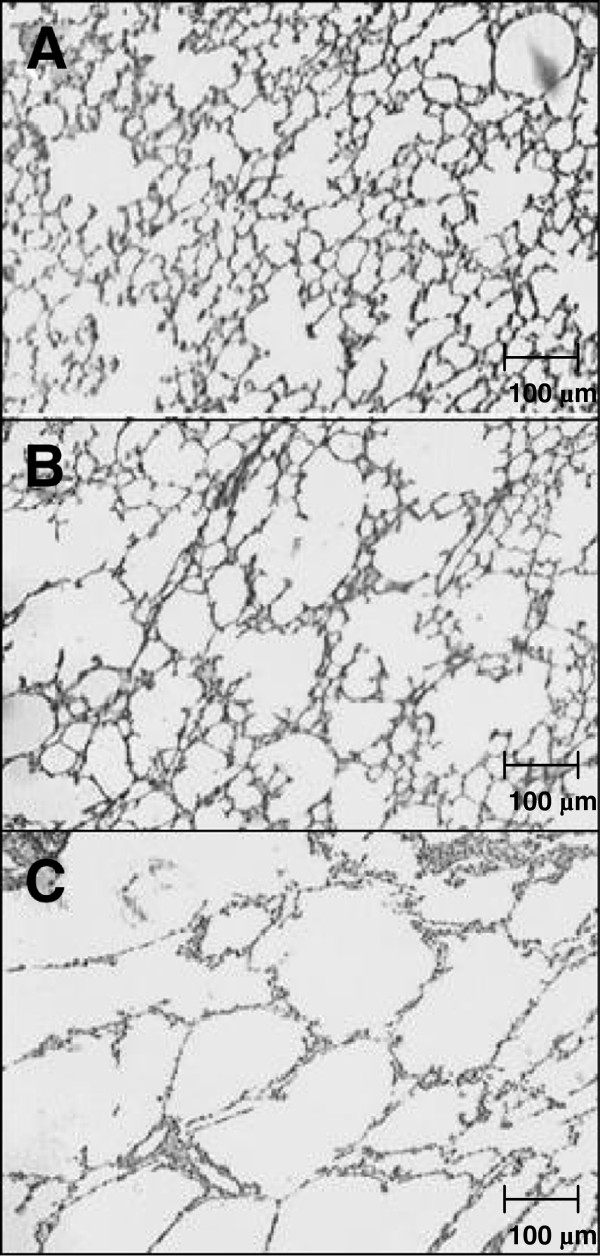
**Histological images from lungs of papain- and saline-treated hamsters. **(**A**) control + saline (CS): animals treated with approximately 0.3 mL of saline only; (**B**) emphysema 20 mg/mL (E20): animals treated with approximately 0.3 mL of 20 mg/mL papain in saline; and (**C**) emphysema 40 mg/mL (E40): animals treated with approximately 0.3 mL of 40 mg/mL papain in saline. Animals were euthanized after 60 days. Hematoxylin and eosin (H & E) images are shown at 50× magnification. Alveolar destruction was determined by the number of times that a predetermined group of coherent lines (1.25 mm^2 ^of total area and 1.50 mm of total length) crossed the parenchymal structures. The lesser these structures are crossed, the more extensive is the lesion.

The extent of lung damage in the emphysema groups was evaluated by the number of crossed alveolar intercepts. No lung injury was detected in the saline-treated group (CS) relative to a control group (no saline or papain instillation). Likewise, no difference was observed in any other parameter that was analyzed in CS animals (data not shown). In contrast, the E20 and E40 groups had significantly decreased crossed intercept values (60.52 ± 2.8 and 52.27 ± 4.7, respectively) as compared to the CS group (94.36 ± 7.3). The comparison between E20 and E40 also demonstrated significant differences in emphysema severity (p < 0.05). Representative images of lung injury are presented in Figure 1. Table 1 shows that the final body weight of CS animals (171.6 ± 15.9 g) increased as compared to the E20 and E40 group animals, which presented decreased values (157.5 ± 10.3 g and 137.37 ± 7.2 g, respectively, p < 0.05 only for the E40 group). The cachexia index (expressed as a percentage) reflects not only the loss of total body weight but also the absence of weight gain [25]. This index in the E20 and E40 groups reduced by 6.28% ± 0.91% and 9.45 ± 0.76%, respectively, as compared to the CS group. The weight of the gastrocnemius muscle was significantly different between the CS (251.87 ± 24.87 mg) and E40 (197.87 ± 10.49 mg) groups, and the E20 (230.12 ± 23.52 mg) and E40 groups. These results represented an 8.6% muscle loss in the E20 group and a 21.4% muscle loss in the E40 group as compared to the gastrocnemius muscle weight of the CS animals.

**Table 1:** Animal and skeletal muscle adaptation characteristics

	**CS**	**E20**	**E40**
**Crossed Alveolar Intercept**	94.36 ± 7.3	60.52 ± 2.8^*^	52.27 ± 4.7^*, †^
**Total body weight (g)**	171.6 ± 15.9	157.5 ± 10.3	137.4 ± 7.2^*, †^
**CI (%)**	---	6.28 ± 0.91	9.45 ± 0.76
**Gastrocnemius weight (mg)**	251.87 ± 24.87	230.12 ± 23.52	197.87 ± 10.49^*, †^
**% of variation on gastrocnemius **(compared with CS)	---	−8.6	−21.4

CS – Group instilled with 300 μL of NaCl 0.9%; E20 – Group instilled with 300 μL of papain 20 mg/mL in NaCl 0.9%; E40 – Group instilled with 300 μL of papain 40 mg/mL in NaCl 0.9%. CI – Cachexia index = (initial body weight – final body weight + body mass gain of CS group) / (initial body weight – body mass gain of CS group) x 100. ^*^p<0.05 when compared with CS and ^†^p<0.05 when compared with E20 by One way ANOVA followed by Bonferroni’s multiple comparison test.

### Oxidative stress

**Figure 2:**
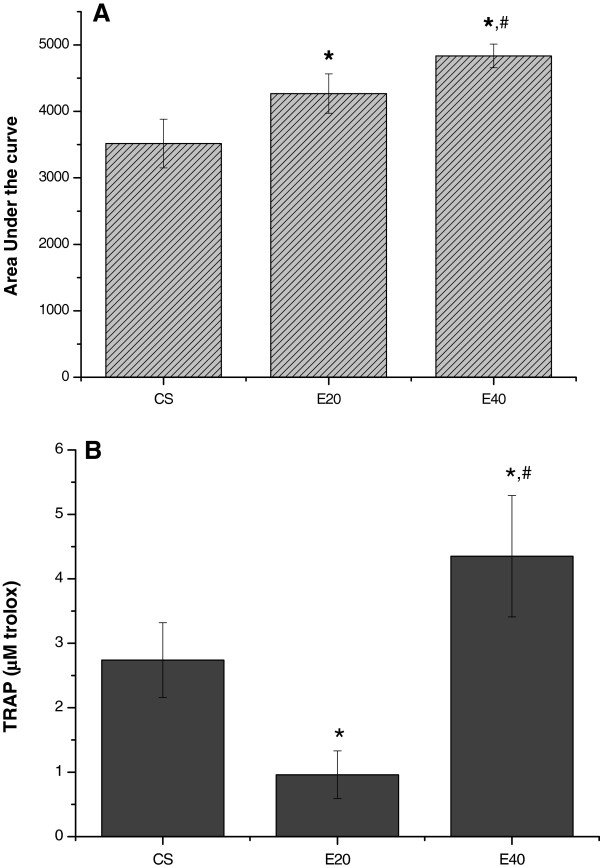
**Effect of muscle loss on gastrocnemius muscle lipid hydroperoxide and antioxidant levels in emphysemic hamsters and controls. **(**A**) Tert-butyl hydroperoxide-initiated chemiluminescence was monitored continuously for 40 min. The area under the curve for each animal was determined for comparisons between groups. (**B**) Total Reactive Antioxidant Potential in gastrocnemius muscles of emphysema and control hamsters. The bars represent the means of 8 animals. Statistical analyses were performed by one-way analysis of variance (ANOVA) followed by Bonferroni’s multiple comparison test, with p < 0.05 considered significant. *p < 0.001 to CS; ^**#**^p < 0.001 relative to E20, as detected by one-way ANOVA followed by Bonferroni’s multiple comparison test.

*Tert*-butyl hydroperoxide-initiated chemiluminescence was used to analyze the integrity of non-enzymatic antioxidant defenses and the levels of lipid hydroperoxides in muscle homogenate of animals inoculated with tumor cells. This assay indicates that the increase in CL is closely related to the oxidative stress previously suffered by the tissue. It induces the consumption of antioxidants and augments the formation of lipid hydroperoxides, which results in increased photon emission [26,31,32]. Figure 2A represents CL areas under the curves extracted from individual 40 min-CL curves of each animal. When E20 and E40 were compared with CS (3517.24 ± 365.40 URL/g tissue), both groups presented increased curves (4268.36 ± 295.63 and 4835.16 ± 178.12 URL/g tissue, respectively, p<0.001 for both). E20 vs. E40 comparison also presented significantly differences. Figure 2B reveals the total antioxidant capacity (TRAP) on gastrocnemius muscles of emphysema and control hamsters. Interestingly, decreased total oxidant capacity on E20 (0.96 ± 0.37 μM trolox) was observed when compared with CS (from 2.74 ± 0.58 μM trolox p<0.05). On the other hand, E40 showed enhancement (4.35 ± 0.94 μM trolox, p<0.05) when it was compared with CS. When E40 was compared to E20, it showed an increase of 353.12%.

**Figure 3:**
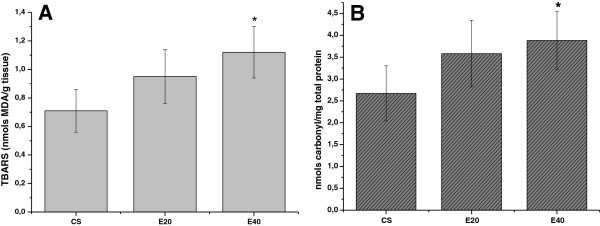
**Effect of emphysema on skeletal muscle in terms of thiobarbituric acid reactive substances (TBARS) and carbonyl proteins in hamsters. **(**A**) TBARS levels in muscle homogenates of hamsters treated with 2 different doses of papain. (**B**) Levels of protein carbonylation in muscle homogenates from control and emphysema hamsters. Results are presented as mean ± standard error of the mean (SEM; n = 8). Statistical differences were detected by one-way ANOVA followed by Bonferroni’s multiple comparison test. Control + saline (CS): animals treated with approximately 0.3 mL of saline only; emphysema 20 mg/mL (E20): animals treated with approximately 0.3 mL of 20 mg/mL papain in saline; and emphysema 40 mg/mL (E40): animals treated with approximately 0.3 mL of 40 mg/mL papain in saline. *p < 0.001 relative to CS, as detected by one-way ANOVA followed by Bonferroni’s multiple comparison test.

Figure 3A shows TBARS levels in the CS, E20, and E40 groups. Only E40 animals (1.12 ± 0.18 nmol MDA/g tissue) showed increased TBARS relative to the CS group (0.71 ± 0.15 nmol MDA/g tissue, p < 0.01). Figure 3B shows the carbonyl protein levels of each group. Similar to the TBARS data, only E40 animals (3.88 ± 0.66 nmol carbonyl/mg total proteins) were significantly different from CS animals (2.67 ± 0.63 nmol carbonyl/mg total proteins,) in terms of carbonyl protein level. No differences were detected between the E20 and E40 groups.

### Chymotrypsin-like activity

**Figure 4:**
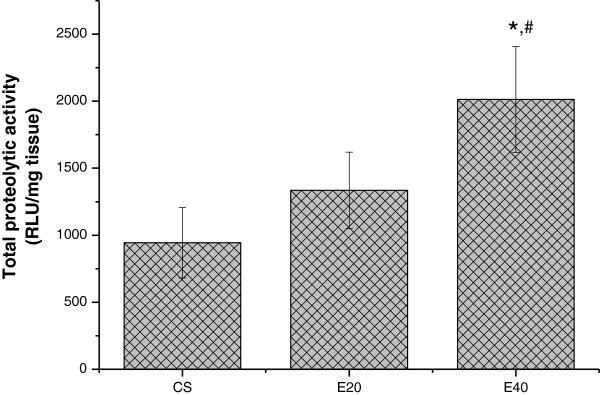
Chymotrypsin-like proteolytic activity was evaluated in the gastrocnemius muscle of hamsters subjected to intratracheal instillation of papain or saline. *p < 0.001 to CS; ^**#**^p < 0.001 relative to E20, as detected by one-way ANOVA followed by Bonferroni’s multiple comparison test.

Increase (113.46%) in the chymotrypsin-like activity was detected in the E40 group (2013.06 ± 394.50 URL/mg tissue) compared with the CS group (943.53 ± 262.52 URL/mg tissue, p < 0.001); a significant increase (50.84%) of chymotrypsin-like activity was also observed in the E40 group as compared to the E20 group (1334.49 ± 285.77 URL/mg tissue, p < 0.001). No significant differences were detected between the CS animals and E20 animals. These results are presented in Figure 4.

### Correlation analysis

As demonstrated in Table 2, the analysis between emphysema severity (represented by the number of crossed alveolar intercepts) and several variables showed high correlation values (body weight = 0.87, muscle weight = 0.72, CL = −0.95, TBARS = −0.98, carbonyl proteins = −0.99, and chymotrypsin-like proteolytic activity = −0.90). In addition, chymotrypsin-like proteolytic activity and the oxidative variables also showed high correlation (CL = 0.97, TBARS = 0.96, and carbonyl proteins = 0.91).

**Table 2:** Sum of correlations presented between emphysema severity, proteolytic activity and oxidative stress variables

	**Body weight loss**	**Gastrocnemius weight**	**TBARS**	**Carbonyl proteins**	**Lipid peroxidation by CL**	***Proteolytic activity***
***Crossed Alveolar Intercept***	0.87	0.72	−0.98*	−0.99*	−0.95*	−0.90*
***Chymotrypsin-like activity***	−0.99*	−0.97*	0.96*	0.91*	−0.97*	-

Mean, SE and N from each group were combined and used in order to establish the correlations between the variables. TBARS – Thiobarbituric acid reactive substances. Positive values mean positive correlation; negative values mean negative correlation, as evaluated by Pearson’s correlation test. * p<0.05.

## Discussion

Emphysema induces human skeletal muscle loss [5], reductions in locomotory skeletal muscle contractile function [33], lipid peroxidation, and alterations in the glutathione redox system in skeletal muscles of hamsters [2]; it also decreases skeletal muscle oxidative enzyme capacity in hamsters [12]. In patients with COPD, there is a relationship between FEV1 (the most acceptable parameter on establishment of severity in humans) and the functional status. Accordingly, a relationship between peripheral muscle strength and the severity of airflow obstruction, if present, would support the idea that chronic inactivity and muscle deconditioning are important in explaining muscle weakness in patients with COPD [33].

Our results showed significantly impaired crossed alveolar intercepts in E20 animals, which was worse in the E40 group. The comparison between E20 and E40 was also significantly different, showing injury enhancement on E40. The damaged lungs are shown in tissue micrographs in Figure 1. The total body weight and gastrocnemius muscle weight were decreased in the E20 group and were statistically significant in the E40 as compared to the CS group. The cachexia index was high in E20 animals, but higher in the E40 group. A positive correlation between emphysema severity and both body and muscle weight indicated a weak relationship between lung damage and cachexia. Bernard et al. [33] suggested that, in patients with stable COPD, the muscle contractile apparatus is preserved due to the fact that loss of muscle mass is proportional to the reduction in strength. The preferential loss in lower limb muscle mass and strength in patients, and their relationship with FEV1 percentage of predicted also suggest that muscle deconditioning and the related disuse atrophy are important factors in explaining peripheral skeletal muscle dysfunction in COPD. Skeletal muscle loss and its relationship with severity are poorly discussed on literature. To our knowledge, these factors were not related with oxidative alterations and their consequences.

Mechanisms underlying muscle wasting observed in several diseases remain largely unknown. Since ROS are demonstrated to be involved on cellular adaptation, recent studies in our laboratory [25] and others [2,15] have demonstrated the involvement of oxidative stress in skeletal muscle loss. In the present study, concomitant muscle wasting and lipid peroxidation indicated that lipid peroxidation might be an important factor in the mechanism of muscle protein hyper-catabolism. Tert-butyl hydroperoxide-initiated CL was originally used to analyze the integrity of non-enzymatic antioxidant defenses and the levels of lipid hydroperoxides in muscle homogenates of animals inoculated with tumor cells. Previous studies using this assay indicated that the increase in CL is closely related to the oxidative stress previously suffered by the tissue. Oxidative stress induces the consumption of antioxidants and augments the formation of lipid hydroperoxides, which results in increased photon emission [26,31]. TRAP on E20 result is in accordance with CL. Increased values of CL area and diminished values of TRAP represent the classic concept of oxidative stress [34]. While CL curves enhance on E20 the total antioxidant capacity diminishes. On the contrary, in E40, CL curves and total antioxidant capacity becomes higher. In respect with this data, Palace et al. [35] demonstrated that the supply of vitamin A to the myocardium by storage organs during increased oxidative stress subsequent to myocardial infarction was showed in hemodynamically assessed rats using compartment analysis of a radio-labeled vitamin A. In our study, it seems that the same phenomenon happens, with a transport of antioxidants stimulated by free radicals damages, which can not handle the deleterious action of radicals at all.

In addition, we observed a strong association between chymotrypsin-like activity and lipid peroxidation markers (CL, TBARS and carbonylated proteins) in this emphysema model. It appears that ROS contribute to skeletal muscle dysfunction in a several ways. For example, Brotto and Nosek [36] demonstrated a blunted Ca^2+^ release from the sarcoplasmic reticulum, and Andrade et al. [37] demonstrated reduced Ca^2+^ sensitivity in skeletal muscles exposed to H_2_O_2_. ROS have been implicated in enzymatic dysfunction within the glycolytic pathway, the citric acid cycle, and the electron transport system, suggesting that elevated ROS may impair cellular energetics within skeletal muscles [38]. In addition, Mattson et al. [2] demonstrated increased lipid peroxidation (evaluated by MDA levels) in gastrocnemius muscles of hamsters with single-dose elastase-induced emphysema; these findings are in agreement with our results. Moreover, we further confirmed the association between oxidative stress and loss of muscle mass and chymotrypsin-like proteolytic activity by using a sensitive CL method [26,31,39], which estimates the chain reaction of lipid peroxidation earlier, i.e., it measures both membrane lipid hydroperoxide formation and antioxidant depletion [40,41]. Our results showed a progressive increase in TBARS and CL, which were strongly correlated with chymotrypsin-like proteolytic activity and increased tissue damage. In addition, progressive protein carbonylation was observed, which was well correlated with chymotrypsin-like proteolytic activity and lung tissue damage. Although Mean, SE and N from each group were combined and used in order to establish the correlations between the variables, there is no difference when all data are put together and the comparison is made point-by-point (with each single animal data put in the analysis as a point), since the calculation of correlation takes in account the mentioned values even when row data is used on the calculus.

A previous study demonstrated that treatment of C_2_C_12_ myotube cells with FeSO_4_/H_2_O_2_ caused a significant rise in MDA levels, with a concomitant increase in the catabolism of myofibrillary proteins and expression of the major components of the ubiquitin-proteasome pathway [42]. The authors suggested that mild oxidative stress increases protein degradation in skeletal muscles by causing upregulation of the ubiquitin-proteasome proteolytic pathway in this *in vitro* model. In line with these findings, in the present study, chymotrypsin-like proteolytic activity was well correlated with CL, TBARS, and carbonyl proteins, and a reduction of body and muscle weight and an increase in emphysema severity were observed. It is worth considering that increased MDA levels are associated with increased proteolysis, which is related to carbonyl proteins levels. It is likely that when MDA or low-molecular-weight adducts is present, the level of oxidized proteins increases, and proteolytic activity could be accelerated, leading to muscle atrophy. Some authors have demonstrated that mild oxidative stress induces protein oxidation, with increased intracellular proteolysis [20,43,44]. Additionally, it has been postulated that mammalian cells are able to selectively remove moderately aldehyde-modified proteins from their intracellular protein pools and that the proteasomal system is responsible for this activity [42]. The 3 major proteolytic activities (chymotrypsin-like, trypsin-like, and post-glutamyl peptide hydrolytic or caspase-like activity), occurring within the 20S core of the 26S proteasome complex are responsible for most of the protein degradation, which includes degradation of damaged cellular proteins.

The bulk of oxidized proteins can be degraded by the proteasomal system [42,45,46], particularly those modified by aldehydes and peroxides [20,43]. Of note, the assay employed in our study can be used to measure proteolysis related to 20S proteasome, and not only the proteasome connected to ubiquitin-marked proteins. The 20S proteasome is also important, as it can degrade oxidized proteins without ubiquitination [44]. Only Debigaré et al. [21] demonstrated that ubiquitination and proteolysis occur in the limb and respiratory muscles of patients with COPD, although no links were established between these processes and the oxidative status.

For the first time, the present study demonstrated that emphysema promotes body weight and skeletal muscle loss in a severity-dependent manner and is related to oxidative stress and chymotrypsin-like proteolytic activity. Additionally, oxidative stress variables and muscle chymotrypsin-like proteolytic activity were well correlated. Thus, it is reasonable to assume that muscle atrophy observed in this model of emphysema is mediated by increased muscle proteolytic activity, with possible involvement of oxidative stress.

## Conclusion

In summary, emphysema induces increased muscle loss, oxidative stress, contractile protein degradation, and chymotrypsin-like proteolytic activity in a lung injury-dependent manner. It is possible that this mechanism can help elucidate the skeletal muscle dysfunction in animal models of emphysema and also in human COPD patients, thus contributing to the establishment of therapeutic countermeasures against emphysema-induced muscle damage and cachexia.

## Abbreviations

COPD: Chronic obstructive pulmonary disease; TBARS: Thiobarbituric acid reactive substances; CL: Chemiluminescence; MDA: Malondialdehyde; ROS: Reactive oxygen species; CS: Control + saline; E20: Emphysema instilled with 20mg/mL of papain; E40: Emphysema instilled with 40mg/mL of papain; i.p: Intraperitoneally; KH_2_PO_4_/K_2_HPO_4_: Monobasic potassium phosphate buffer; KCl: Sodium chloride; TBA: Thiobarbituric acid; RT: Room temperature; DNPH: Dinitrophenyhydrazine; HCl: Chloridric acid; TCA: Trichloroacetic acid; RLU: Relative light units; BSA: Bovine serum albumine; SEM: Standart error mean; H_2_O_2_: Hydrogen peroxide; FeSO_4_: Ferrous sulfate.

## Competing interests

Authors declare that they have no competing interests.

## Authors’ contributions

JT carried out CL and MDA assays, and drafted the manuscript. ALC participated in the design of the study and perfomed the statistical analysis. CRB carried out carbonyl proteins assays and helped with CL data treatment. SSB carried out MDA and carbonyl protein data treatment, analysis and interpretation. RC was responsible for critical review and intellectual content. FAG conceived the study, and participated in its design and coordination, and helped to draft the manuscript. All authors read and approved the final manuscript.

## Pre-publication history

The pre-publication history for this paper can be accessed here:

http://www.biomedcentral.com/1471-2474/14/39/prepub
